# The Diabetic Lung Can Be Ameliorated by *Citrullus colocynthis* by Reducing Inflammation and Oxidative Stress in Rats with Type 1 Diabetes

**DOI:** 10.1155/2023/5176645

**Published:** 2023-07-21

**Authors:** Mohammad Abbas Bejeshk, Fatemeh Bagheri, Fouzieh Salimi, Mohammad Amin Rajizadeh

**Affiliations:** ^1^Department of Physiology, Faculty of Medicine, Kerman University of Medical Sciences, Kerman, Iran; ^2^Pathology and Stem Cell Research Center, Department of Pathology, Afzalipour Medical Faculty, Kerman University of Medical Sciences, Kerman, Iran; ^3^Legal Medicine Research Center, Legal Medicine Organization, Kerman, Iran; ^4^Department of Clinical Biochemistry, School of Medicine, Kerman University of Medical Sciences, Kerman, Iran; ^5^Physiology Research Center, Institute of Neuropharmacology, Kerman University of Medical Sciences, Kerman, Iran; ^6^Cardiovascular Research Center, Institute of Basic and Clinical Physiology Sciences, Kerman University of Medical Sciences, Kerman, Iran

## Abstract

**Background:**

Diabetes impacts various organs in the body and some reports showed that the lung is also affected by diabetes, and an imbalance of inflammation and oxidative stress may participate to diabetic lung impairments. The present study is conducted to assess the impacts of *Citrullus colocynthis* (CC) on some aspects of these impairments.

**Methods:**

Frothy two male Wistar rats (3-4 months old and weighing 200–250 g) were used in the present research. Animals were divided into 3 groups of control, Diabetes, and Diabetes + Drug. CC was administered to diabetic rats orally. The lung tissue and BALF oxidative stress and inflammatory indices including the MDA, TAC, SOD, Gpx, TNF*α*, IL-6, IL-17, and IL-10 were evaluated by the ELISA method.

**Results:**

Our observations disclosed the ameliorative impacts of CC administration against oxidative stress and inflammation imbalance. Also, it was found that CC improved body weight and fasting blood sugar in rats with diabetes.

**Conclusion:**

We can conclude that the administration of CC can be effective in improving diabetic lungs in rats.

## 1. Introduction

Type 1 diabetes or Diabetes mellitus (DM) is a systemic malady represented by a chronic hyperglycemic circumstance and inflammation and oxidative stress are serious consequences of this disease [1]. Based on the data released by World Health Organization (WHO), about 442 million persons suffered from DM in 2014. The estimates indicated that the number of diabetic patients in the world will raise to 592 million persons by 2035 [2]. The main side effects of DM are caused by its microangiopathic and macroangiopathic symptoms, affecting the eyes, kidneys, nerves, heart, major vessels, and lungs [3]. These complications mainly result from vascular damage that plays a vital pathophysiological role in diabetes [4]. The link between lung dysfunction and diabetes is assumed to be the outcome of biochemical alterations developed in the pulmonary and respiratory structures. These components involve a set of mechanisms potentially caused by hypoxemia, oxidative stress, systemic inflammation, or direct damage due to chronic hyperglycemia [5–7]. Impaired lung performance is explored in more than 73% of diabetic patients [8]. Inadequate glucose control can lead to lung dysfunction and elevated systemic levels of inflammatory cytokines [9]. TNF-*α* (Tumor necrosis factor *α*) can mediate the diabetic process and in association with Oxidative stress may impair the metabolism of glucose [10]. Scientific data suggest that diabetes involves inflammatory changes in the lung [11]. Additionally, serum levels of IL-6 (Interleukine-6) in T2DM patients disrupt endothelial cell function [12]. Experimental data also disclosed that IL-6 plays a vital role in developing inflammatory diseases in the respiratory system [13]. Recent studies have found a link between islet amyloid deposition and islet inflammation. Accordingly, islet amyloid involves in producing inflammatory cytokines mainly interleukin-1*β* (IL-1 *β*) by macrophages and dendritic cells [14]. Oxidative stress is one of the factors accounting for pulmonary alterations [15]. An increasing bulk of studies proposes that hyperglycemia and high oxidants accumulation induced by free fatty acid (FFA) can more easily impair the function of *β*-cells due to antioxidant (SOD (Superoxide dismutase), CAT (Catalase), and GPx (Glutathione peroxidase)) subnormal expression in *β*-cells [16]. Oxidative stress may also lead to lung endothelial impairment in diabetic patients [17–19]. Traditional plants are widely used for treating DM [20]. *Citrullus colocynthis* (CC) (melon) belonging to the Cucurbitaceae family [21] produces significant levels of antioxidant phenolics and flavonoids [22]. The seeds of this plant are used for their medicinal effects, such as soothing, antioxidant, and anti-inflammatory properties [21]. Despite the growing number of clinical studies on the diabetic lung, less attention has been paid to the mechanism accounting for diabetes-induced pulmonary disorders. Thus, the current investigation designed to assess the effects of the aqueous extract of CC seeds on glycemic, inflammatory cytokines, TNF-*α*, IL-6, IL-1*β*, and IL-10 levels, and oxidative status by detecting SOD, GPx activity, TAC (Total antioxidant capacity), NO (Nitric oxide), and MDA (Malonyl dialdehyde) levels in tissue and BALF (Broncho alveolar lavage fluid) in rats with T1DM.

## 2. Materials and Methods

### 2.1. Crude Extract Preparation

The method of crude extract preparation of CC was shown in our previous study [23]. Briefly, CC fruits were gathered from the Ilam province in Iran and after removing of their seeds, the seeds dried for 72 h. These seeds are a rich source of some fatty acids. In addition, the seeds are rich in essential amino acids and minerals. We grounded 100 gram of seeds using by a mixer and we added the grounded seeds to 1 liter of distilled water. The mixture was heated for 2 h at 80°C. After that the extract was passed through Whatman No. 1 filter paper. At the end of extraction, the fractions obtained were gathered in a balloon and lyophilized, yielding the lyophilized aqueous extract [23–25].

### 2.2. Animals

All protocols and treatments carried out in this were confirmed (Ethics code: IR.KMU.REC.1398.127) by Kerman University of Medical Sciences. We tried to provide maximum comfort to the animals at all research procedures. The age and weight of animals used in this experiment were 3-4-month-old and 200–250 gr respectively. They were kept under controlled conditions. 42 male wistar rats were assigned to the three groups: The healthy rats (Control group), The rats receiving STZ (Diabetes group), and The diabetic rats receiving CC extraction orally (Diabetes + Drug group).

### 2.3. Induction of Diabetes

The type 1 diabetes model was developed after overnight fasting (8 hours). The induction of severe diabetes was performed in the animals by a single STZ dose (50 mg/kg body weight). Moreover, STZ was prepared in sodium citrate buffer at pH 4.5 and administered intraperitoneally with insulin syringes [26]. Type 1 diabetes symptoms such as severe urination was recorded four days after injection. However, to ensure diabetes, fasting blood sugar (FBS) was measured after 15 days. The 40-day curation started on the 15th day after the induction of diabetes [23].

### 2.4. Drug Administration

15 days after the injection of STZ, the remediation began with CC. Thus, the aqueous extract of CC (200 mg/kg) was administered orally every day for 40 consecutive days [27]. A dose response study was conducted to reaching the dose with maximum effectiveness. The effective dose was the dose that had the best effect in reducing blood sugar. The rats received glucose (2 g/kg body weight) and two different doses of Citrullus-colocynthis aqueous extract (CCAE) (100 or 200 mg/kg body weight). The average blood glucose was 80–126 mg/kg. Blood glucose was measured every 30, 60, 90, and 120 minutes in a blood drop taken from the tail by a glucometer. The CCAE dosage of 200 mg/kg was more effective than 100 mg/kg in correcting the two-hour postprandial blood sugar levels. Consequently, the diabetic rats in all the treated groups received CCAE (200 mg/kg) as the daily oral treatment [23]. Also, other authors such as Kalva et al. used a dose of 200 mg/kg [27].

### 2.5. BALF Collection

After sacrificing, BALF collected from the left lungs and centrifugation accomplished instantly (10 min, 4°C, 1000 g) and the supernatant was utilized to measurement the inflammatory and oxidative factors levels [28–32].

### 2.6. Biochemical Evaluations

The Bradford method utilized for total proteins assessment. Inflammatory and oxidative parameters were measured in tissue supernatant and BALF. The exact methods for evaluation of oxidative parameters were mentioned in our previous study [29]. Briefly, The level of the SOD enzyme was evaluated using a colorimetric assay. GPx activity was measured by reducing cumene hydroperoxide. Malondialdehyde (MDA), was assessed using the TBARS method. Total antioxidant capacity (TAC) was evaluated by the FRAP assay. In addition, nitrite, total protein and all oxidant and anti-oxidant parameters were evaluated based on the kit's instructions (Navand Salamat Co., Iran). Quantitative investigations of cytokines were accomplished using the double-antibody Sandwich enzyme linked immunosorbent assay (ELISA) kits based on the manufacturers' instructions (Karmania Pars gene Co., Iran) [33–36].

### 2.7. Lung Injury Score

The right lungs and airways were gathered from rats and immersed in 10% formalin. Then, the hematoxylin/eosin (H&E) was utilized for tissues staining. The histopathological scoring was performed based on our previous study [34].

### 2.8. Statistical Analyses

The data are reported as a mean ± SEM. Normality was checked using by Shapiro Wilk test. One-way ANOVA followed by Tukey's post hoc test, used for data analyzing. Significant level was considered at *p* < 0.05 [37, 38].

## 3. Results

### 3.1. The Impacts of CC Administration on Body Weight in Rats with Diabetes

**Figure 1:**
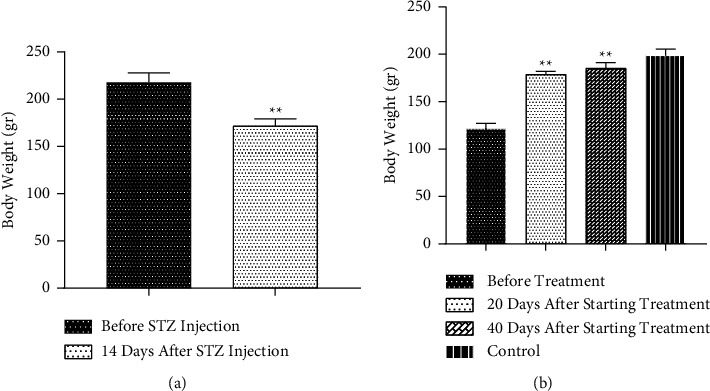
The impacts of diabetes and CC on body weight (a) The comparison between diabetic and nondiabetic animals (paired *T*-test used for statistical analysis) and (b) The impacts of CC on body weight in different times (repeated measurement used for statistical analysis). Mean ± SEM, (^*∗∗*^) *p* < 0.01 vs before STZ injection, as shown in Figure 1(a). (^*∗∗*^) *p* < 0.01 vs before starting treatment, as shown in Figure 1(b).

In the Diabetes and Diabetes + Drug (before commencing therapy) groups, diabetic rats' body weight considerably dropped (*p* < 0.01). Additionally, repeating research experiments into body weight in the Diabetes + Drug group after the commencement of treatment revealed that the body weight considerably raised after 20 and 40 days of CC administration compared to before beginning treatment (*p* < 0.01). The treatment and control groups did not vary statistically significantly (Figures 1(a) and 1(b)).

### 3.2. The Impacts of CC on FBS in Rats with Diabetes

**Figure 2:**
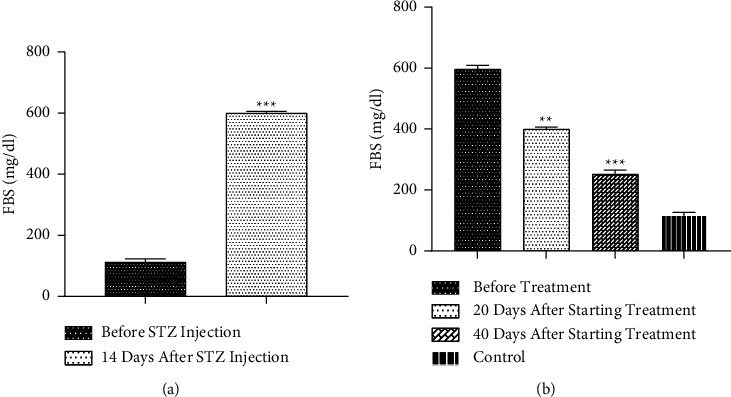
The impacts of diabetes and CC on FBS (a) The comparison between diabetic and nondiabetic animals (paired *T*-test used for statistical analysis) and (b) The impacts of CC on FBS in different times (repeated measurement used for statistical analysis). Mean ± SEM, (^*∗∗∗*^) *p* < 0.001 vs before STZ injection, as shown in Figure 2(a). (^*∗∗*^) *p* < 0.01 & (^*∗∗∗*^) *p* < 0.001 vs before starting treatment, as shown in Figure 2(b).

In rats with diabetes in the Diabetes and Diabetes + Drug (before commencing therapy) groups, when compared to 14 days before to STZ administration, the FBS of rats dramatically raised (*p* < 0.001). Additionally, measurement of FBS after beginning therapy revealed that in the Diabetes + Drug group, the administration of CC for 20 and 40 days resulted in a marked reduction in FBS as compared to baseline (*p* < 0.01). No significant difference between the treatment groups and the control group was found after repetition-based analysis (Figures 2(a) and 2(b)).

### 3.3. The Impacts of CC on Oxidative Stress and Protein Leakage in Lung and BALF

**Figure 3:**
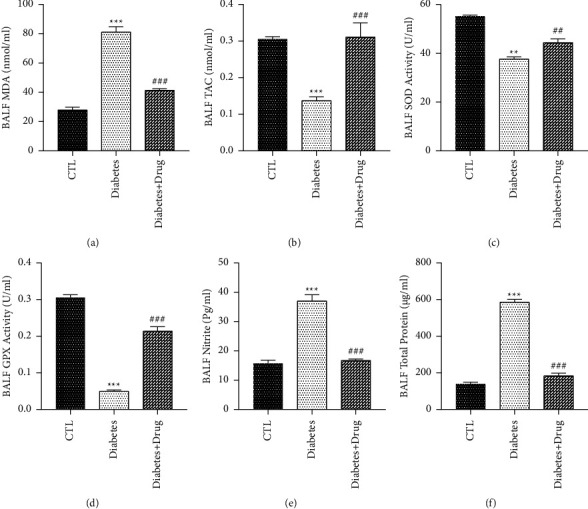
The impacts of diabetes and CC on BALF oxidant and anti-oxidants and total protein levels. Mean ± SEM, (^*∗∗∗*^) *p* < 0.001 and (^*∗∗*^) *p* < 0.01 vs control group. (^###^) *p* < 0.001 and (^##^) *p* < 0.01 vs diabetes group.

**Figure 4:**
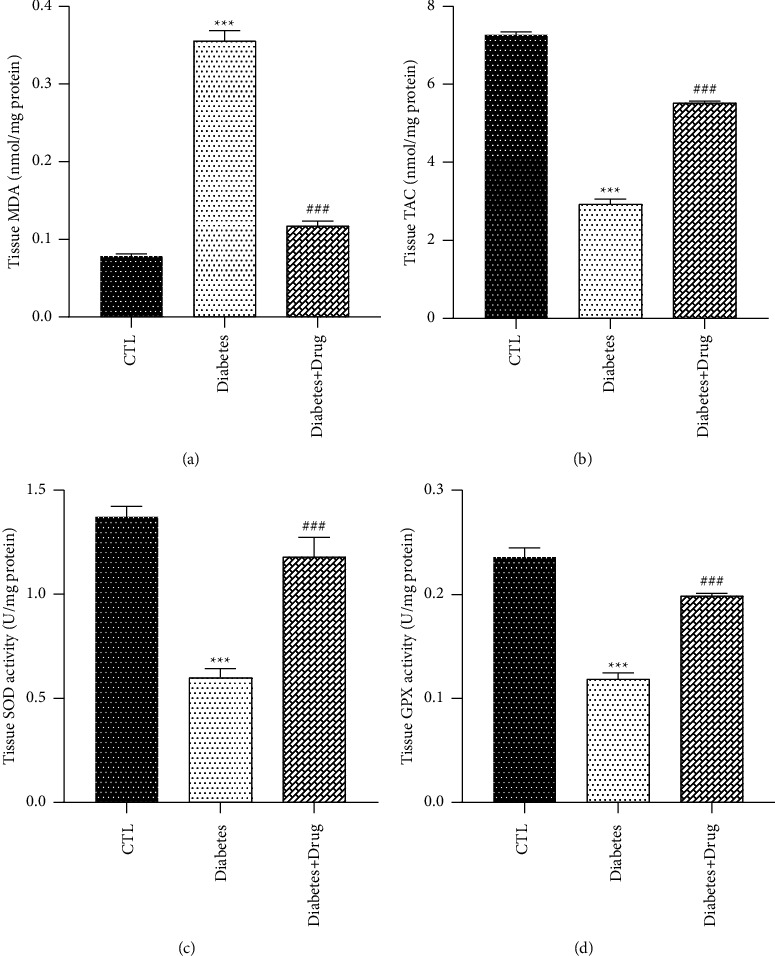
The impacts of diabetes and CC on lung tissue oxidant and anti-oxidants levels. Mean ± SEM, (^*∗∗∗*^) *p* < 0.001 vs control group. (^###^) *p* < 0.001 vs diabetes group.

The results revealed that NO level in BALF and MDA level in tissue and BALF elevated in the diabetic rats in comparison with the healthy rats (*p* < 0.001), and remediation with CC resulted in a significantly decrease the MDA and NO levels (*p* < 0.001). In the rats with diabetes, meaningful decreased SOD activity was observed in tissue and BALF (*p* < 0.001, *p* < 0.01; respectively) as well as GPx activity and TAC level in tissue and BALF was remarkably reduced (*p* < 0.001) in comparison with the healthy rats but was remarkably elevated after treatment with CC (*p* < 0.001). There was an elevation (*p* < 0.001) in the total protein content of the BALF of the T1D animals compared with control subjects. Treatment with CC reduced the protein leakage in the diabetic animals (*p* < 0.001) (Figures 3 and 4).

### 3.4. The Impacts of CC on Cytokines

**Figure 5:**
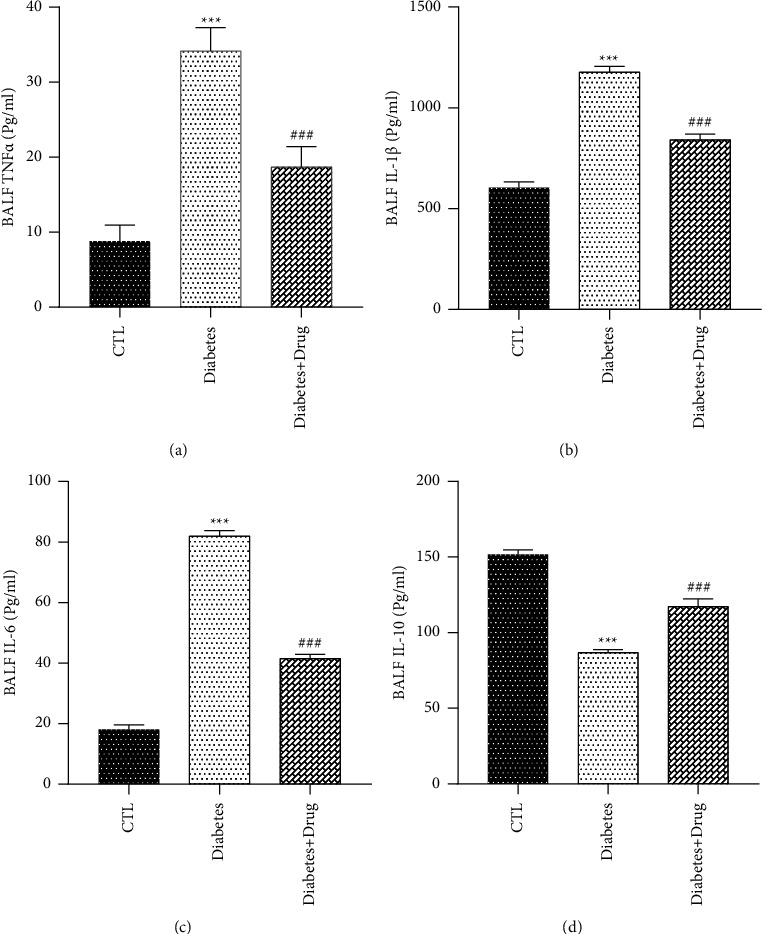
The impacts of diabetes and CC on BALF inflammatory and anti-inflammatory cytokines levels. Mean ± SEM, (^*∗∗∗*^) *p* < 0.001 vs control group. (^###^) *p* < 0.001 vs diabetes group.

**Figure 6:**
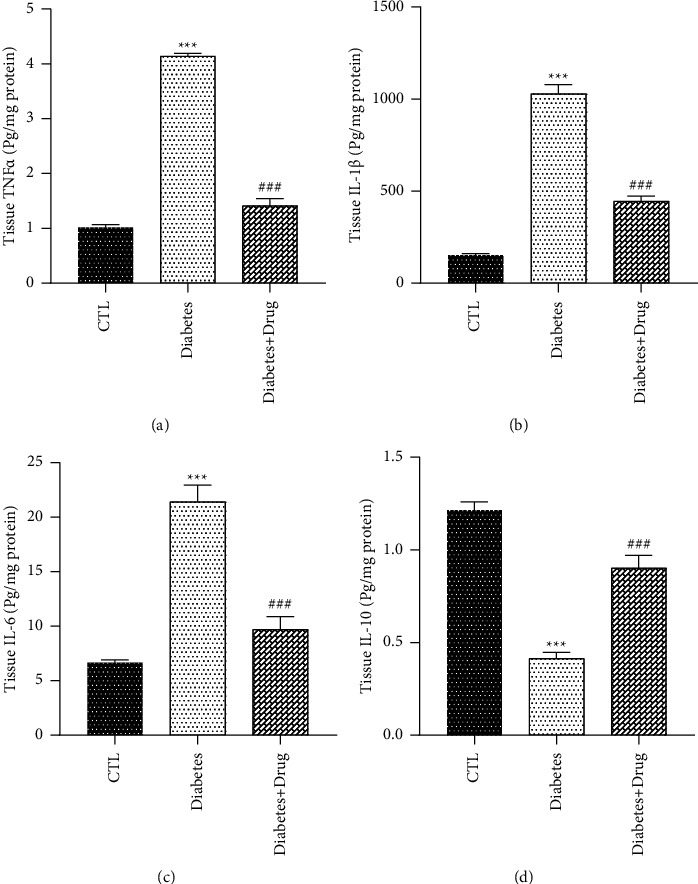
The impacts of diabetes and CC on lung tissue inflammatory and anti-inflammatory cytokines levels. Mean ± SEM, (^*∗∗∗*^) *p* < 0.001 vs control group. (^###^) *p* < 0.001 vs diabetes group.

The BALF and tissue TNF-*α*, IL-6, and IL-1*β* levels in diabetic rats were more than Group Control, and those levels were significantly lower after CC treatment than those in group diabetes (*p* < 0.001) in comparison with the healthy rats, the IL-10 level was reduced in group diabetes, and significant increase were found in the group receiving the CC (Figures 5 and 6).

### 3.5. Lung Injury Scores

**Figure 7:**
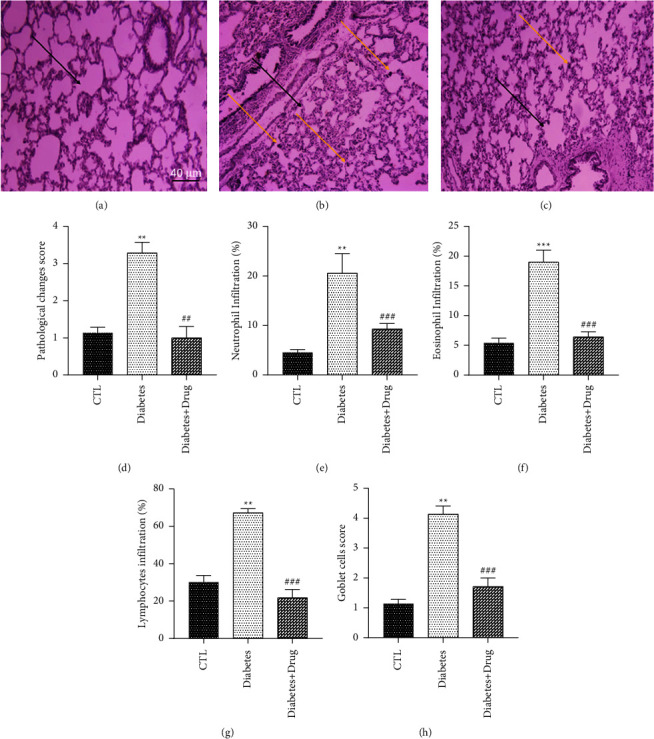
Micrographs of the lung stained with H&E (a–h). (a) Control: (b) diabetes (c) diabetes + drug (d) pathological changes score (e) neutrophil infiltration (%) (f) eosinophil infiltrations (g) lymphocyte infiltrations (h) goblet cells score. Black arrows indicate alveolar collapse and congestion status. Yellow arrows indicate inflammation. Data are presented as mean ± SEM. (^*∗∗∗*^) *p* < 0.001 and (^*∗∗*^) *p* < 0.01 vs control group. (^###^) *p* < 0.001 and (^##^) *p* < 0.01 vs diabetes group.

The pathological injury score elevated in the diabetic rats in comparison with the control group and curation with CC remarkably diminished this damaged condition in comparison with the diabetic rats. Infiltration of neutrophils, eosinophils and lymphocytes elevated in the peribronchial and perivascular area of diabetic group in comparison to the control group. These factors were also considerably alleviated by CC. The number of goblet cells elevated in the lungs of diabetic rats in comparison with the control animals. This factor remarkably diminished in groups cured with CC in comparison with the diabetes group (Figure 7).

## 4. Discussion

Mechanisms associated with chronic hyperglycemia can induce oxygen radicals formation and systemic inflammation in diabetic patients [9, 39]. The main contribution of this research was that diabetes enhances the lung injury and inflammation in rats due to elevated levels of TNF-*α*, IL-6, and IL-1*β* and lower levels of IL-10, lower SOD and GPx activity, TAC levels, and increased MDA and NO levels in tissue and BALF. Moreover, the diabetic lung went through marked histological changes leading to high lung injury scores. Traditional medicinal plants have been long used all over the world for a wide range of diabetic symptoms [17]. In this study, the aqueous extract of CC seeds was used to treat diabetes, and the data conformed the improvement in lung status diminished impairments in rats with diabetes.

As demonstrated in the current research, inflammatory cytokines remarkably elevated in the lung of the rats with diabetes. Moreover, inflammatory cell infiltration such as neutrophil, eosinophil, and lymphocyte infiltration was significantly correlated with high lung pathological change scores. In contrast, the protective mechanisms including IL-10 declined in diabetic rats. Dennis et al. found lower lung function in diabetic subjects. This decline can be attributed to elevated levels of inflammatory cytokines such as TNF-*α*, as confirmed in the current research [9]. Xiong et al. demonstrated that the increased levels of TNF-*α* and IL-6 can act as indices of an inflammation [40]. Hu et al. reported elevated TNF-*α* expression with diabetes [41]. The present study revealed that lung injury was accompanied by early increase in TNF-*α*, IL-1*β*, and IL-6 in tissue and BALF. Our data demonstrated that treatment of rats with CC declined the TNF-*α*, IL-1*β*, and IL-6 levels in comparison with the nontreated rats, and increased IL-10, suggesting that increased IL-10 levels may shift the balance in favor of an anti-inflammatory condition. A review of the literature did not find any studies exploring the immunoinflammatory response of CC against the diabetic lung. However, previous studies have addressed the effectiveness of CC in other diseases. Sanadgol et al. found that the CC lowered the levels of TNF-*α* and IL-6 while maintaining the IL-10 level in overweight rats [42]. Pashmforosh et al. also reported that the CC cream suppresses TNF-*α* and IL-6 [43].

Histological evidence confirmed the infiltration of neutrophils, eosinophils, and lymphocytes in the lung of the diabetic group. Histopathological data in diabetic patients confirmed thickened alveolar, epithelial, and pulmonary capillary basal lamina [4]. Regarding with the findings of the current research, Zhang et al. confirmed the histological alterations in the diabetic lung after 8 weeks of diabetes in rats [16]. Kolahian et al. also confirmed the infiltration of mononuclear cells and edema in the submucosa of the trachea and lung of diabetic animals [2]. These results confirmed the inflammation in the lungs of diabetics. Furthermore, our findings indicated CC reduced inflammatory cell infiltration and pathological change scores and eventually decreased airway damage score. Hoffmann et al. found that bitter apple is a useful remediation for treating inflammation in a colitis model [44].

Mucins mainly secretes from airway goblet cells and leading to the formation of a mucus layer that protects the epithelium. The data in present study showed that the number of goblet cells enhanced substantially in the lung of diabetic rats. Besides, treatment with CC significantly declined mucus hypersecretion and goblet cell hyperplasia, demonstrating the ameliorative impacts of CC against lung injury. Hoffmann et al. found that bitter apple treatment could restore the number of goblet cells, indicating the tissue-healing capacity of the phytochemical [44].

The data from animal experiments demonstrated that experimental T1DM impairs pulmonary vascular endothelial function via oxidative stress [2]. Similarly, the data in the present study revealed an elevation in lung oxidative stress in diabetic rats compared the controls, and a decline in antioxidant enzyme SOD and GPx activity and TAC level. These findings conformed the results reported by other authors, who showed an increase in oxidative stress in the lungs of diabetic rats compared to the controls. They also reported a reduction in the antioxidant enzyme SOD activity [15]. Furthermore, Xiong et al. showed the increased MDA level, a lipid peroxidation marker, associated with the depressed activity of SOD, as the most significant endogenous anti-oxidase in diabetic lung injury [40]. Gumieniczek et al. reported the intensified levels of the lipid peroxidation process and lower activities of antioxidative compounds in the diabetic lung [45]. Endothelial NO is a remarkable factor in vascular function. Diabetes can lead to an increment of this molecule. Our data demonstrated this increment in diabetic lungs. The present study confirmed significantly lower GPx and SOD scavenger and TAC levels in diabetes, but an increase in CC. Our data also confirmed that CC treatment can significantly reduce the production of free radicals as demonstrated by the ameliorated MDA and NO levels. Rizvi et al. also found that CC acts against oxidative stress [46]. Likewise, Ostovan et al. demonstrated the beneficial effects of CC against oxidative stress in the diabetic rats [47]. These findings further confirmed the antioxidative effect of CC as a useful mechanism in diminishing lung damage in diabetics.

Overall, the data in the current research revealed the protective effect of CC on the diabetic lung in rats. CC can reduce inflammatory cell infiltration into lung tissues. The data also showed that the administration of CC can prevent oxidative stress and inflammation in BALF and lung tissue. Thus, a further exploration of such medicines might offer a natural key to finding new anti-diabetic drugs.

## Data Availability

The datasets used and/or analyzed during the current study are available from the corresponding author on reasonable request.
